# The Effect of Direct and Indirect EZH2 Inhibition in Rhabdomyosarcoma Cell Lines

**DOI:** 10.3390/cancers14010041

**Published:** 2021-12-23

**Authors:** Andreas Schmidt, Lucas Behrendt, Jana Eybe, Steven W. Warmann, Sabine Schleicher, Joerg Fuchs, Evi Schmid

**Affiliations:** 1 Department of Pediatric Surgery and Pediatric Urology, University Children’s Hospital, Eberhard Karls University Tuebingen, Hoppe-Seyler-Strasse 3, 72076 Tuebingen, Germany; lucasbehrendt@me.com (L.B.); jana.eybe@student.uni-tuebingen.de (J.E.); steven.warmann@med.uni-tuebingen.de (S.W.W.); joerg.fuchs@med.uni-tuebingen.de (J.F.); evi.schmid@med.uni-tuebingen.de (E.S.); 2 Department of Pediatric Hematology and Oncology, University Children’s Hospital, Eberhard Karls University Tuebingen, Hoppe-Seyler-Strasse 1, 72076 Tuebingen, Germany; sb.schleicher@outlook.de

**Keywords:** rhabdomyosarcoma, RH30, RD, EZH2, epigenetic, EPZ005687, DZNep, AdOx

## Abstract

**Simple Summary:**

Rhabdomyosarcoma is the most common soft tissue tumor in children. Its two major subtypes show epigenetic alterations that are associated with poor prognosis. Therefore, targeting these epigenetic alterations by pharmacological intervention could be a therapeutic approach. We investigated two different types of substances that interfere with the epigenetic process of histone methylation. We performed studies in two cell lines that carry characteristics of the major rhabdomyosarcoma subtypes. The aim of this study was to find out if the substances differ in their effect on tumor-related cellular functions and to find out if the tumor subtypes differ in their response to the substances. These findings may contribute to a better assessment of the feasibility of pharmacological intervention directed against histone methylation in subtypes of rhabdomyosarcoma.

**Abstract:**

Enhancer of Zeste homolog 2 (EZH2) is involved in epigenetic regulation of gene transcription by catalyzing trimethylation of histone 3 at lysine 27. In rhabdomyosarcoma (RMS), increased EZH2 protein levels are associated with poor prognosis and increased metastatic potential, suggesting EZH2 as a therapeutic target. The inhibition of EZH2 can be achieved by direct inhibition which targets only the enzyme activity or by indirect inhibition which also affects activities of other methyltransferases and reduces EZH2 protein abundance. We assessed the direct inhibition of EZH2 by EPZ005687 and the indirect inhibition by 3-deazaneplanocin (DZNep) and adenosine dialdehyde (AdOx) in the embryonal RD and the alveolar RH30 RMS cell line. EPZ005687 was more effective in reducing the cell viability and colony formation, in promoting apoptosis induction, and in arresting cells in the G1 phase of the cell cycle than the indirect inhibitors. DZNep was more effective in decreasing spheroid viability and size in both cell lines than EPZ005687 and AdOx. Both types of inhibitors reduced cell migration of RH30 cells but not of RD cells. The results show that direct and indirect inhibition of EZH2 affect cellular functions differently. The alveolar cell line RH30 is more sensitive to epigenetic intervention than the embryonal cell line RD.

## 1. Introduction

Epigenetic dysregulation may be involved in the development, growth, and metastasis of a variety of tumors [1,2]. Rhabdomyosarcoma (RMS) show increased abundance of Enhancer of Zeste Homologue 2 protein (EZH2) [3,4,5,6], a catalytic subunit of the polycomb repressive complex 2 (PCR2), and altered epigenetic markers compared to skeletal muscle [7,8].

RMS is the most common soft tissue tumor in children originating from mesenchymal precursors and expressing features of early myogenic differentiation [9]. Based on histology and genetics different subtypes can be distinguished [10,11,12]. The most common histological subtypes are the embryonal and alveolar subtype which account for approximately 60% and 20% of RMS [13,14]. More important for prognosis than histology is the translocations t(2;13)(q35;q14) and t(1;13)(p36;q14), which occur in 55% and 22% of alveolar RMS [15]. They result in the expression of *PAX3:FOXO1* and *PAX7:FOXO1* fusion proteins and are associated with poor prognosis [15,16]. The embryonal RMS is characterized by the loss of heterozygosity at the 11p15.5 locus, different chromosomal gains and losses, and somatic mutations [17,18]. Fusion-negative alveolar RMS share cellular features such as the loss of heterozygosity and clinical characteristics including good prognosis with the embryonal RMS [19]. The cell lines RH30 and RD are developed from a metastasized alveolar RMS and a recurrent embryonal RMS, respectively [20,21]. Although substantial achievements in the therapy of RMS have been made in the past decades, the overall survival rate of patients with high-risk and metastasized RMS amounts only to about 30% [22,23].

The abundance of EZH2 protein in RMS and its association with poor prognosis, increased metastasis and lymph node involvement make EZH2 an interesting therapeutic target [24,25,26]. Different approaches to affect EZH2 have been investigated [27]. Direct inhibitors of the methyltransferase activity of EZH2 such as EPZ005687 bind to the catalytic S-adenosylmethionine (SAM) pocket of the SET (Su(var)3-9, Enhancer of Zeste, Trithorax) domain of EZH2 [28,29]. Indirect inhibitors such as 3-deazaneplanocin (DZNep) and adenosine dialdehyde (AdOx) inhibit the S-adenosyl-L-homocysteine (SAH) hydrolase, which leads to increased SAH levels and a feedback inhibition of methyltransferase activity [30,31,32]. Indirect inhibitors reduce not only the methyltransferase activity of EZH2 but also globally of enzymes that use S-adenosyl-L-methionine (SAM) as a methyl donor [32,33]. In addition, they decrease EZH2 protein levels [31,32] and thus may affect the canonical activity of methylating H3K27 and also multiple noncanonical activities of EZH2 such as activating or inhibiting transcription factors [34,35]. Therefore, the cellular effects of indirect and direct inhibitors may differ.

It was shown that the direct inhibitor MC1945 and DZNep reduce the proliferation and xenograft growth of RD and RH30 cells but differ in inducing apoptosis [4,5]. We investigated and compared the effect of the direct inhibitor EPZ005687 and the indirect inhibitors DZNep and AdOx on a variety of cellular functions which characterize tumor behavior such as cell viability, colony formation, migration, apoptosis, cell cycle, and spheroid size and viability. The aim of this study was to characterize the two types of inhibitors in more detail to better assess the feasibility of this therapeutic approach by these types of inhibitors in the two major RMS subtypes. 

## 2. Materials and Methods

### 2.1. Cell Lines and Reagents

The embryonal RMS cell line RD (ATCC, Manassas, VA, USA) and the alveolar RMS cell line RH30 (DSMZ, Braunschweig, Germany) as well as the primary human skeletal muscle cells (SkMC) (PromoCell, Heidelberg, Germany) were cultured in DMEM high glucose 4.5 g/L medium (Sigma Aldrich Chemie GmbH, Taufkirchen, Germany) supplemented with 1% L-glutamine (Biochrom, Berlin, Germany), 10% heat-inactivated fetal bovine serum (Biochrom, Berlin, Germany), and 1% penicillin/streptomycin (Biochrom, Berlin, Germany) in a humidified atmosphere containing 5% CO_2_ at 37 °C. Only early passages (after purchase or authentication) which were tested to be negative for mycoplasma contamination (MycoAlert; Lonza, Cologne, Germany) were used for the current study. EZH2 inhibitors EPZ005687 (Cayman Chemical Company, Ann Arbor, MI, USA), DZNep (Cayman Chemical Company, MI, USA), and AdOx (Sigma Aldrich Chemie GmbH, Taufkirchen, Germany) were used as indicated.

### 2.2. Western Blotting

EZH2 protein abundance in RMS cell lines, treated with EPZ005687, DZNep, and AdOx for 24 h was detected by Western blotting as previously described [36]. The membranes were incubated with primary rabbit EZH2 antibody (1:1000, Cell Signaling Technology, Inc. (Danvers, MA, USA), New England Biolabs (Ipswich, MA, USA), 98 kDa) overnight at 4 °C. Incubation with rabbit monoclonal GAPDH antibody (1:1000, Cell Signaling Technology, Inc., New England Biolabs) served as a loading control.

### 2.3. Cell Viability Assay

In a humidified atmosphere containing 5% CO_2_ at 37 °C, 8 × 10^3^ (RH30) or 1.5 × 10^4^ (RD and SkMC) cells were seeded in 96-well plates in a final volume of 100 μL culture medium per well. After overnight adherence of the cells, 72 h treatment with EPZ005687, DZNep, and AdOx was started. Every 24 h, the medium and treatment drug was renewed. The viability assays were performed as previously described [37]. 

### 2.4. Clonogenic Assay

RMS cancer cell lines were plated in 6-well plates at 750 cells per well. After incubation with or without EPZ005687, DZNep, and AdOx for 72 h, cells were washed twice with PBS and fresh medium was added. The colonies grew for 7–10 days before being fixed with 99.9% methanol for 5 min and stained with 1% (*w*/*v*) crystal violet for 30 min for RD cells, and 20 min for RH30 cells at room temperature. Images were captured using a phase-contrast microscope Zeiss Axiovert 135 microscope (original magnification, ×5; Carl Zeiss Microscopy GmbH, Jena, Germany). The number of colonies (>50 cells) was counted microscopically [38]. Dividing the number of colonies by the number of plated cells and multiplying by 100 yielded the colony formation rate according to Franken et al. [39].

### 2.5. Wound Healing Assay

For would healing assay, 6 × 10^5^ cells per well were plated onto 12-well dishes. A single scratch wound was inflicted using a sterile micropipette tip in each confluent monolayer. Cells were washed with PBS to remove cell debris and incubated with or without EPZ005687, DZNep, and AdOx for 72 h (every 24 h medium and treatment substance were renewed) and monitored by photographs directly after the scratch was performed and 24 h later. Images (three per well) were captured using a Zeiss inverted microscope (Axiovert 135) with a 10× objective lens and Canon EOS 550D digital camera. The wound width was measured using Axio-Vision 3.1 Software and expressed as a percentage of the initial wound width. 

### 2.6. Flow Cytometry

Apoptosis assays of RD and RH30 cancer cell lines were analyzed using flow cytometry with Annexin V staining with allophycocyanin (APC) conjugation (BD Biosciences, Franklin Lakes, NJ, USA) and propidium iodide (PI) staining after incubation in the presence or absence of EPZ005687, DZNep, and AdOx for 72 h with a renewal of medium and treatment substance every 24 h.

After incubation, the adherent cells were collected and stained with Annexin V (BioLegend, Koblenz, Germany)/Propidium Iodide (Sigma Aldrich Chemie GmbH, Taufkirchen, Germany) in Annexin Binding Buffer according to the manufacturer’s recommendations. Acquisition and analysis of data were conducted with a BD FACS CANTO II flow cytometer and FACS Diva Software Version 8.0 (Becton Dickinson, Heidelberg, Germany).

### 2.7. Spheroids

For the spheroids, 2 × 10^5^ RD and RH30 cells per well in 100 µL previously filtered (Easytrainer^TM^ 40 µm, Greiner Bio-One GmbH, Kremsmünster, Austria) cell culture medium were pipetted onto a low-attachment round bottom 96-well plate (Thermo Fisher Scientific, Waltham, MA, USA). Centrifugation at 200× *g*, 5 min at room temperature and incubation for 72 h allow the cells to form spherical aggregates. The cancer cells were treated in the presence or absence of EPZ005687, DZNep, and AdOx for 72 h. Every 24 h, a change in treatment was performed, during which only 50 µL of the cell culture medium was aspirated. The growth behavior of the spheroids was documented photographically every 24 h. With the AxioVision 3.1 software, the change in the spheroid size was analyzed. Finally, the spheroids were incubated with 25 µL methylene blue per well for 24 h and measured on the Multiple Plate Reader (Victor X, PerkinElmer Inc., Waltham, MA, USA) with the wavelengths 486 nm and 535 nm (fluorescein). The analysis of the data was performed with GraphPad Prism.

### 2.8. Cell Cycle

For cell cycle, 4 × 10^6^ cells (RD) and 3 × 10^6^ cells (RH30) per well were plated onto 6-well dishes. After attachment, cells were incubated over night with a temperature of 37 °C, 95% humidity, and 5% CO_2_. Cancer cells then were treated with EPZ005687, DZNep, and AdOx for 72 h. Every 24 h, a change in treatment was performed (during which only 50 µL of the cell culture medium was aspirated). After resuspension, cells were transferred into a Neubauer chamber and were counted. The lowest cell number was noted. For staining, for all cells, this number was used, and the corresponding volume was transferred into a FACS tube. All tubes were filled with medium to the same volume. Centrifugation at was performed at 1.500 U/min, 5 min at room temperature, with a tilt overhang. Cells were washed twice with 2 mL of cold BPS and then centrifuged. After the second wash, the supernatant was removed, and the tubes were placed on ice. Then, the tubes were carefully resuspended and fixed with 1 mL of ice-cold 80% ethanol for 30 min on ice. Again, cells were washed twice with cold PBS and separated by centrifugation. Finally, cells were incubated with 500 µL FXCycle PI/Rnase staining solution (Invitrogen, Waltham, MA, USA) per tube at room temperature for 20 min. The acquisition of data was conducted with a BD FACS CANTO II flow cytometer (Becton Dickinson, Heidelberg, Germany) and analysis was performed with FlowJo software.

### 2.9. Statistical Analysis

GraphPad Prism 8 (GraphPad Software, La Jolla, CA, USA) was used for statistical analyses. All data were tested for significance using ANOVA (Dunnett correction). Only results with *p* ≤ 0.05 were considered statistically significant. Data are presented as means ± standard error of the mean (SEM) unless otherwise specified. All data are representative of at least three experiments.

## 3. Results

### 3.1. EZH2 Protein Abundance Is More Reduced by Indirect Inhibitors Than by the Direct Inhibitor

**Figure 1:**
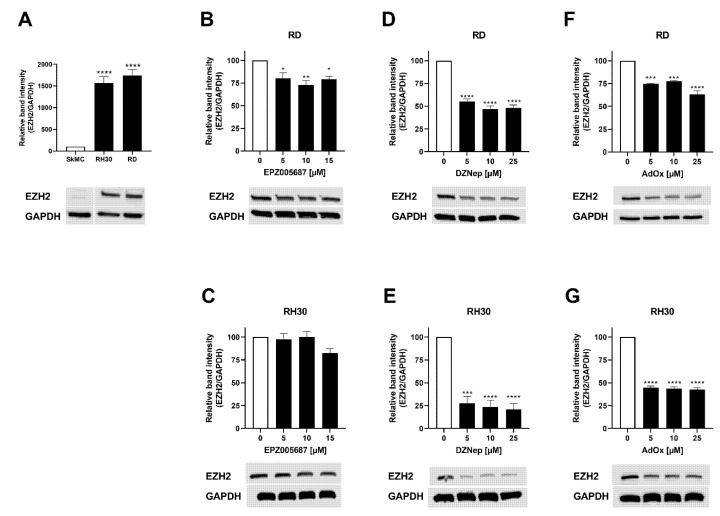
EZH2 abundance in RMS cell lines in the presence or absence of EZH2 inhibitors. (**A**) Representative Western blot of EZH2 and densitometric quantification of SkMC, RD, and RH30 cells. Relative ratio of EZH2/GAPDH density was normalized to ratio obtained in SkMC. Effect of EPZ005687 (**B**,**C**), DZNep (**D**,**E**), and AdOx (**F**,**G**) on RD (upper row) and RH30 cells (lower row) on EZH2 abundance. Relative ratio of EZH2/GAPDH density was normalized to ratio obtained in untreated control cultures. GAPDH was used as loading control. Error bars represent mean +/− SEM (*n* = 3). *p* * ≤ 0.05, *p* ** ≤ 0.01, *p* *** ≤ 0.001, *p* **** ≤ 0.0001 indicates statistical significance.

The Western blots revealed significantly higher EZH2 protein abundance in the RD and RH30 cell line than in the primary skeletal muscle cells (SkMC). The indirect inhibitors DZNep and AdOx significantly reduced the EZH2 protein abundance in both cell lines at all concentrations tested, more in the RH30 cell line than in the RD cell line. DZNep was more effective in both cell lines than AdOx. A significant decrease in EZH2 abundance in the range of 20–27% by EPZ005687 was detected in RD cells after 72 h of treatment. Only at a high concentration of 15 µM was a non-significant decrease in EH2 abundance observed in RH30 cells (Figure 1). The uncropped Western blots of EZH2 abundance in RMS cell lines in the presence or absence of EZH2 inhibitors can be found in the Supplementary Materials S1. 

In summary, the indirect inhibitors (DZNep and AdOx) have a stronger impact on EZH2 protein abundance in RMS cell lines than the direct inhibitor EPZ005687.

### 3.2. Both Types of Inhibitors Reduced RMS Cell Viability in Both Cell Lines

**Figure 2:**
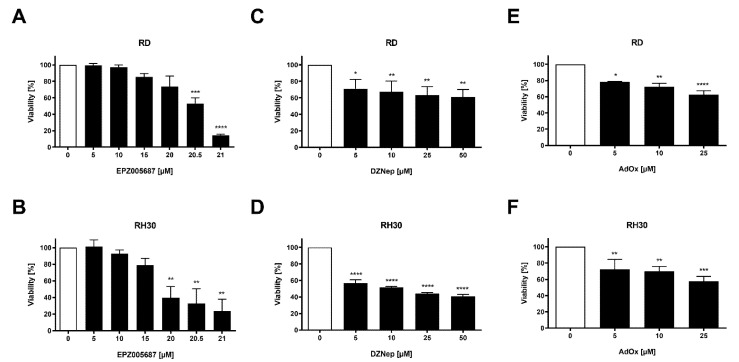
Effect of the EZH2 inhibitors on cell viability in RMS cell lines. Relative numbers of viable RD (upper row) and RH30 (lower row) cells following a 72 h incubation in the absence (white bars) and presence (black bars) of increasing concentrations of EPZ005687 (**A**,**B**), DZNep (**C**,**D**), and AdOx (**E**,**F**). The untreated control was set as 100%. Error bars represent mean +/− SEM (*n* ≥ 3). *p* * ≤ 0.05, *p* ** ≤ 0.01, *p* *** ≤ 0.001, *p* **** ≤ 0.0001 indicates statistical significance.

The reduction of cell viability by EPZ005687 reached significance at a concentration of 20.5 µM and 20 µM in the RD and RH30 cell line, respectively. It was 86% in the RD cell line and 76% in the RH30 cell line at the highest concentration of 21 µM EPZ005687. The reduction of cell viability by DZNep and AdOx was significant at 5 µM in both cell lines (range 22–43%). It increased slightly with increasing concentration but not as strongly as when treated with EPZ005687. At 25 µM, the reduction by DZNep was 37% in the RD and 56% in the RH30 cell line, and by AdOx 45% and 42%, respectively (Figure 2). From these results, it can be concluded that much lower concentrations of the two indirect inhibitors (DZNep and AdOx) can affect the viability of RD and RH30 cells than of EPZ005687.

### 3.3. Migration Was Inhibited by Both Types of Inhibitors in the RH30 Cells but Not in the RD Cells

**Figure 3:**
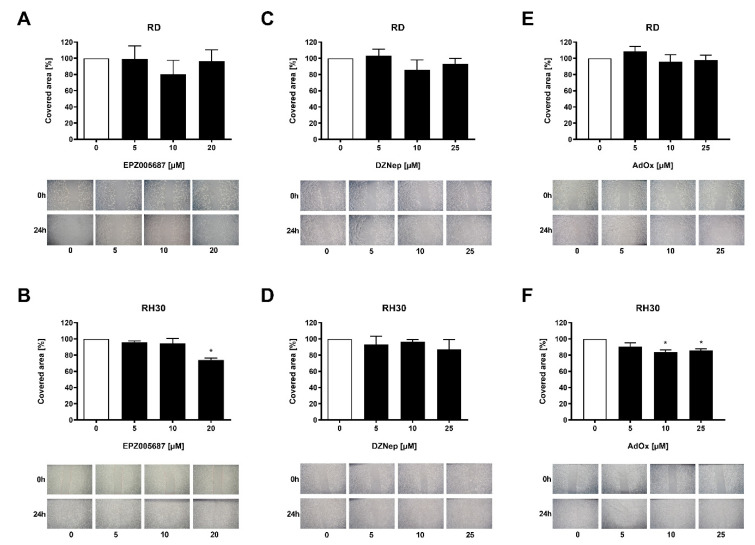
Migration behavior of RMS cells in response to EZH2 inhibitors. A wound healing assay was performed to assess migration. Relative numbers of migrated RD (upper row) and RH30 (lower row) cells following a 72 h incubation in the absence (white bars) and presence (black bars) of increasing concentrations of EPZ005687 (**A**,**B**), DZNep (**C**,**D**), and AdOx (**E**,**F**). The untreated control was set as 100%. Error bars represent mean +/− SEM (*n* = 4). *p* * ≤ 0.05 indicates statistical significance. A representative image of the wound healing assay is shown for each concentration of the inhibitors.

The migration of cells may indicate their potential to spread and metastasize. We investigated the effect of the inhibitors on migration using the wound healing assay. In the RH30 cell line, 20 µM EPZ005687 decreased the migration significantly by 26%, and 10 µM and 25 µM AdOx significantly by 16% and 14%, respectively. The inhibition of 13% achieved by DZNep did not reach statistical significance. Neither of the substances impaired cell migration of RD cells (Figure 3). The data indicate that both types of inhibitors can inhibit migration in RH30 cells but not in RD cells.

### 3.4. Both Types of Inhibitors Reduced Colony Formation of RD and RH30 Cells

**Figure 4:**
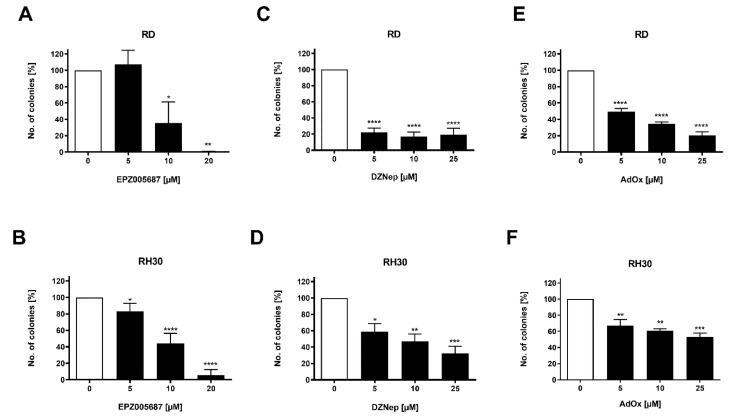
Effect of the EZH2 inhibitors on colony formation. Arithmetic means ± SEM (*n* = 3) of the percentage of evolving clones of RD (upper row) and RH30 (lower row) cells following a 72 h incubation in the presence (black bars) of EZH2 inhibitors EPZ005687 (**A**,**B**), DZNep (**C**,**D**), and AdOx (**E**,**F**) relative to the clones in the absence of the inhibitors (white bars). The untreated control was set as 100%. *p* * ≤ 0.05, *p* ** ≤ 0.01, *p* *** ≤ 0.001, *p* **** ≤ 0.0001 indicates statistical significance.

A further characteristic of cells which is linked to the ability to metastasize is colony formation. All three inhibitors reduced the colony formation concentration-dependently. In both cell lines, the inhibition was stronger by EPZ005687 than by DZNep or AdOx and stronger in RD cells than in RH30 cells (Figure 4). In comparison to cell viability (Figure 2), colony formation was in general more impaired, e.g., for 10 µM EPZ005687, which had no effect on cell viability but showed a significant effect on colony formation at this concentration in both cell lines. DZNep and AdOx also had a more pronounced effect on colony formation than on cell viability. Obviously, colony formation is more sensitive to EZH2 inhibition than cell viability.

### 3.5. Apoptosis Was More Induced by the Inhibitors in the RH30 Cell Line Than in the RD Cell Line

**Figure 5:**
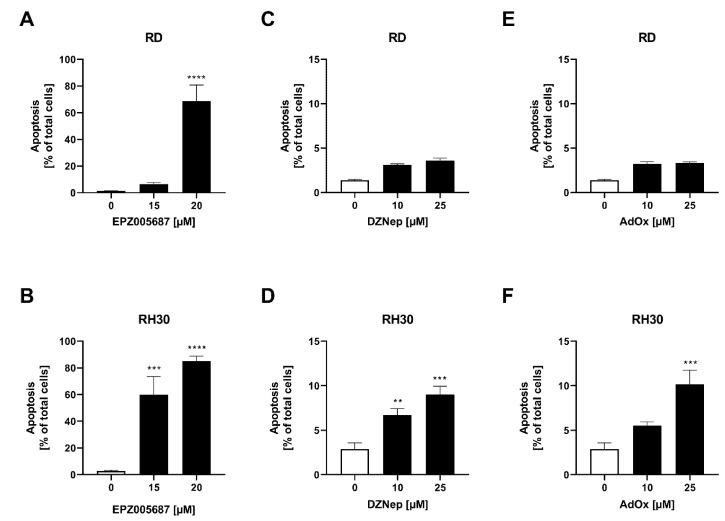
Flow cytometric analysis of apoptosis in RMS cells after treatment with EZH2 inhibitors. Arithmetic means ± SEM (*n* = 3) of the number of Annexin V-positive cells after 72 h incubation with EPZ005687 (**A**,**B**), DZNep (**C**,**D**), and AdOx (**E**,**F**) in RD cells (upper row) and in RH30 (lower row) cells. The untreated control was set as 100%. *p* ** ≤ 0.01, *p* *** ≤ 0.001, *p* **** ≤ 0.0001 indicates statistical significance.

In the apoptosis assay, a significant dose-dependent increase in apoptosis was detected in RH30 cells after 72 h of treatment with the inhibitors. In the RH30 cell line, 20 µM EPZ005687 increased apoptosis significantly to 85%, 25 µM DZNep and AdOx significantly to 9% and 10% of total cells, respectively. In RD cells, 20 µM EPZ005687 increased apoptosis significantly to 59% of total cells. DZNep and AdOx showed a small increase which, however, did not reach statistical significance (Figure 5).

### 3.6. The Direct Inhibitor but Not the Indirect Inhibitors Arrested RD and RH30 Cells in the G1 Phase

**Figure 6:**
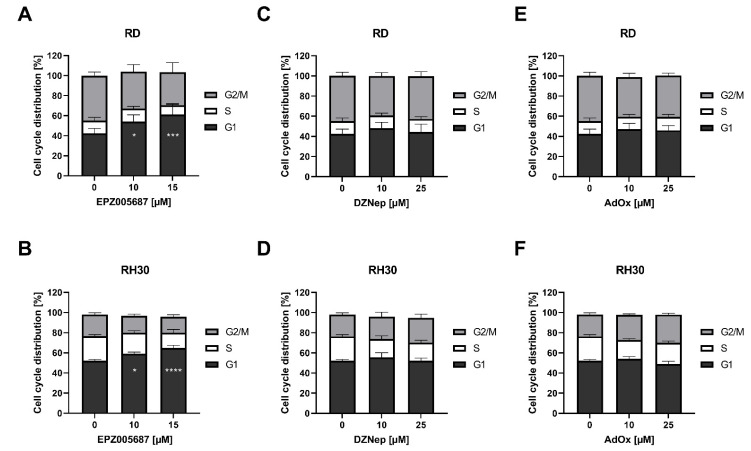
Flow cytometric analysis of cell cycle in RMS cell lines. Effect on cell cycle arrest following a 72 h incubation in the presence (black bars) or absence (white bars) of EPZ005687 (**A**,**B**), DZNep (**C**,**D**), and AdOx (**E**,**F**) on RD (upper row) and RH30 cells (lower row). Error bars represent mean +/− SEM (*n* = 4). *p* * ≤ 0.05, *p* *** ≤ 0.001, *p* **** ≤ 0.0001 indicates statistical significance.

We further investigated the effect of 72 h of treatment with the inhibitors on the distribution of cells within the cell cycle. The proportion of cells in the G1 phase in untreated RD cells and untreated RH30 cells was 42% and 52%, respectively. This proportion did not change significantly under treatment with DZNep or AdOx in either the RD cell line or the RH30 cell line. However, EPZ005687 had a significant concentration-dependent effect in both cell lines. At 15 µM EPZ005687, a further 20% of the cell population of RD cells and 13% of the cell population of RH30 cells were arrested in the G1 phase (Figure 6). 

### 3.7. DZNep Decreased Spheroid Viability and Reduced Spheroid Circumference

Spheroids more closely resemble the three-dimensional situation in tumors than two-dimensional cell cultures. As cell–cell interactions and the microenvironment may modulate cellular functions, spheroids can provide additional information on the susceptibility of cells to pharmacological intervention. We therefore investigated the effect of the inhibitors of EZH2 on spheroid viability and circumference.

**Figure 7:**
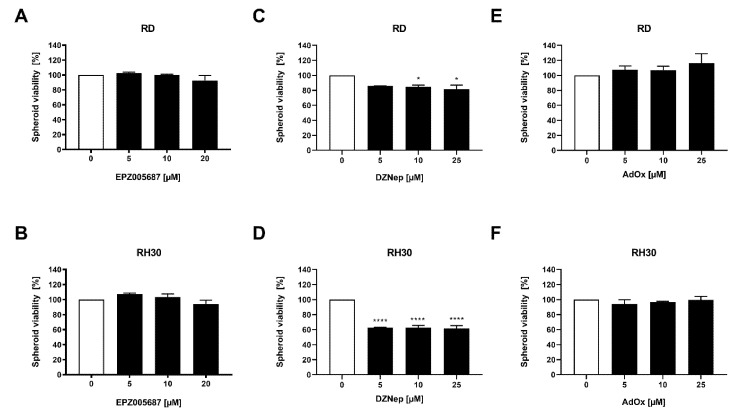
Modulation of EZH2 inhibitors on spheroid viability. Relative numbers of viable RD (upper row) and RH30 (lower row) cells following a 72 h incubation with increasing concentrations of EPZ005687 (**A**,**B**), DZNep **(C**,**D**), and AdOx (**E**,**F**)**.** The untreated control was set as 100%. Error bars represent mean +/− SEM (*n* = 3). *p* * ≤ 0.05, *p* **** ≤ 0.0001 indicates statistical significance.

**Figure 8:**
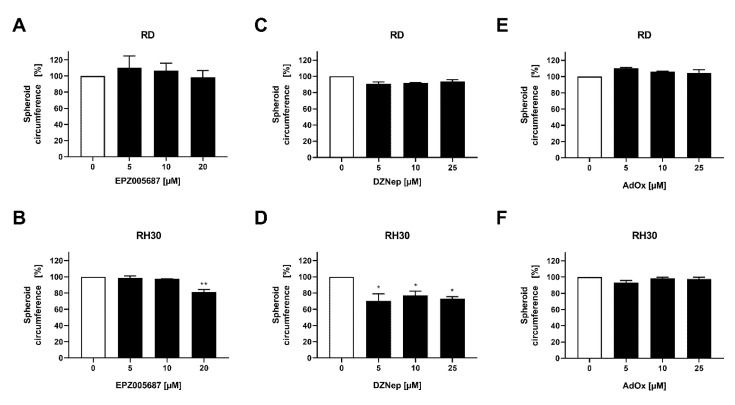
Effect of the EZH2 inhibitors on spheroid circumference in RMS cell lines. Inhibitory effect of EPZ005687 (**A**,**B**), DZNep (**C**,**D**), and AdOx (**E**,**F**) on RD (upper row) and RH30 (lower row) cells on spheroid circumference. The untreated control was set as 100%. Error bars represent mean +/− SEM (*n* = 3.) *p* * ≤ 0.05, *p* ** ≤ 0.01 indicates statistical significance.

DZNep reduced the spheroid viability significantly in the RD cell line by 18% and in the RH30 cell line by 38%. In contrast, neither EPZ005687 nor AdOx impaired the spheroid viability in any cell line (Figure 7). In RD cells, the reduction of spheroid circumference at different DZNep concentrations was about 8% but not significant, whereas in RH30 cells, the reduction of 25% was significant. AdOx had no effect on the spheroid circumference in any cell line. EPZ005687 reduced the circumference at the concentration of 20 µM by 19% (Figure 8).

In the studies on cell viability in two-dimensional cell cultures (Figure 2), EPZ005687 and DZNep showed a comparable inhibition pattern as in the studies on spheroid viability: EPZ005687 exerted no effect in RD cells up to 20 µM and RH30 cells up to 15 µM, and DZNep had an effect even at low concentrations in both cell lines. Contrary to the similarity in the effect of EPZ and DZNep on cell viability and spheroid viability in both cell lines, AdOx had no effect on the spheroid viability, although it impaired the cell viability at all concentrations tested. 

## 4. Discussion

In the present study, we investigated and compared direct and indirect inhibitors of the catalytic activity of EZH2 in the embryonal RMS cell line RD and the alveolar RMS cell line RH30. The results demonstrate that the two types of inhibitors differ in the extent to which they affect cellular functions, and the two cell lines differ in their response to the inhibitors.

The effect of the two types of inhibitors may be related to their mode of action. Both types inhibit the methyltransferase of EZH2. However, indirect inhibitors additionally inhibit SAM-dependent methyltransferases which methylate DNA, lysine residues other than H3K27, and arginine residues in histones [32,33]. Additionally, they reduce the EZH2 abundance and affect other non-canonical activities of EZH2, which include the methylation of nonhistone substrates, protein and microRNA binding, and interactions with transcription factors [34,35]. The significance of methyltransferases other than H3K27 methyltransferase of EZH2 has been shown in chondrosarcoma cell lines. In these cells, DZNep effects were correlated to SAH hydrolase inhibition but not to EZH2 expression or reduced H3K27me3 methylation. The authors conclude that other methyltransferases than EZH2 are involved in the DZNep effect [40]. The significance of non-canonical activities of EZH2 is further demonstrated for castration-resistant prostate cancer cells in which the oncogenicity of EZH2 is related to its role as transcription factor [41,42].

In various cell lines, inhibition of the EZH2 methyltransferase did not copy the reduction in EZH2 abundance [43,44,45,46]. Selective EZH2 degraders have been developed to reduce EZH2 protein. In EZH2-dependent triple-negative breast cancer cells, the newly developed EZH2 specific degrader MS1943 reduced cell proliferation and induced apoptosis effectively in contrast to selective inhibitors of the enzymatic activity of EZH2 [46].

The RH30 cell line and RD cell line carry characteristics of the fusion positive and fusion negative RMS subtype, respectively. The subtypes differ in DNA and histone methylation [47], micro-RNA expression, and gene expression [48,49,50,51], including genes of signaling pathways which are involved in complex cellular functions such as motility, invasion, and metastasis [52,53]. Any difference in response of the cell lines to interference with EZH2 function may be attributed to the differing signal network established in the cells.

In our experiments, migration was inhibited in the RH30 cell line but not in the RD cell line. Regulation of invasion and metastasis involves various signaling pathways and is subject to epigenetic regulation [53]. PAX3-FOXO1 fusion protein available in RH30 cells affects different downstream targets which are involved in cell motility and invasion [54], and it interacts with epigenetic modifiers to regulate transcriptional activities which affect multiple cellular processes including motility [55,56]. The promoter of the GEFT gene is hypomethylated in RH30 cells, leading to an increased activity of the GEFT-Rho-GTPase signaling pathway, which is involved in the regulation of cell motility [57]. Genes regulating cell motility such as the gene of the transcription factor F0XF1 and LMO4 [52], cell motility gene 1 (ELMO1) and NEL-like 1 gene (NELL1) are overexpressed in the RH30 cell line compared to the RD cell line [52,58]. Silencing the genes had a greater effect on migration in the RH30 cell line than in RD cells. The MET receptor is overexpressed in RH30 cells [59,60,61] and may activate the ERK/MAPK pathway [61] which is involved in regulating cell migration. Activating the MET/ERK2 pathway by the hepatocyte growth factor stimulated cell motility of alveolar RMS but not embryonal RMS cells [60]. The SNAIL transcription factor is overexpressed in RH30 cells compared to RD cells [62]. SNAIL is regulating cellular processes including migration by interacting with the PI3K/AKT signaling pathway and micro-RNA [63]. In summary, the RH30 cell line showed marked changes in the network controlling motility compared to RD cells, which may explain their response to the inhibitors.

The spheroid size and viability showed a better response to DZNep, which was not to be expected considering the results of the studies on two-dimensional cell culture. This indicates the limitations of the study. Obviously, epigenetic mechanisms are particularly influenced by cellular interactions so that data of models more complex than two-dimensional models may provide more reliable results. The specific inhibitor MC1945 reduced the growth of xenografts of RD cells, and MC1945 and DZNep reduced the growth of xenografts of RH30 cells in mice [4,5]. Data on more cellular function in complex models and in vivo studies could be helpful to better assess whether a therapeutic application of EZH2 inhibitors could be reasonable.

Ciarapica et al. investigated the direct inhibitor MC1945 and the indirect inhibitor DZNep. She showed that at the concentration of 5 µM of both substances, MC1945 had no effect on EZH2 protein abundance except DZNep, that both types of inhibitors inhibit migration in RH30 cells but not in RD cells and induce apoptosis in RH30 cells but not in RD cells [4,5]. A strict comparison of these findings with our findings faces some limitations as the direct inhibitors EPZ005687 and MC1945 have differing properties such as inhibition constants, as the incubation period in the reported apoptosis experiments was 24 h longer than in our experiments, and as we used higher concentrations of the substances for the apoptosis experiment. Despite these limitations for comparison, it may be concluded that the results of the two studies are in good agreement. Our results also do not indicate an effect on migration and apoptosis at a concentration of 5 µM in RD cells but do in the RH30 cells. The EZH2 protein abundance of RD cells and RH30 cells is reduced by DZNep in both studies. However, whereas both studies show no effect of MC1945 or EPZ005687 on EZH2 protein abundance in RH30 cells, EPZ005687 but not MC1945 decreased it by a small amount in RD cells. It has been shown in cell lines of various tumors that direct inhibitors did not reduce EPZ protein abundance. So, the use of EPZ005687 in our experiments instead of MC1945 seems rather not to explain the different result we observed. 

## 5. Conclusions

In RMS, direct and indirect inhibitors of EZH2 differ in the effects they exert on cellular functions, which may be due to multiple effects by indirect inhibitors. Differences also exist between the embryonal RD cell line and the alveolar RH30 cell line in their response to the inhibitors due to divergence in epigenetic features, gene expression and signaling pathways which are involved in the regulation of cellular functions. It may be concluded that the alveolar and embryonal RMS subtypes are different entities regarding EZH2 inhibition. Cellular interactions seem to influence epigenetic processes as different results of two- and three-dimensional culture systems indicate. Therefore, data of two-dimensional models should be supported by data from more complex models and in vivo studies. 

## Data Availability

The data presented in this study are available in this article.

## Supplementary Materials

The following is available online at https://www.mdpi.com/article/10.3390/cancers14010041/s1, Material S1: Uncropped Western blots of EZH2 abundance in RMS cell lines in the presence or absence of EZH2 inhibitors. Addition to Figure 1.
