# Supplementary material for: The Effect of Direct and Indirect EZH2 Inhibition in Rhabdomyosarcoma Cell Lines
Source: Cancers (Basel). 2021 Dec 23;14(1):41. doi: 10.3390/cancers14010041 (PMC8750739; doi:10.3390/cancers14010041)

Figure S1A

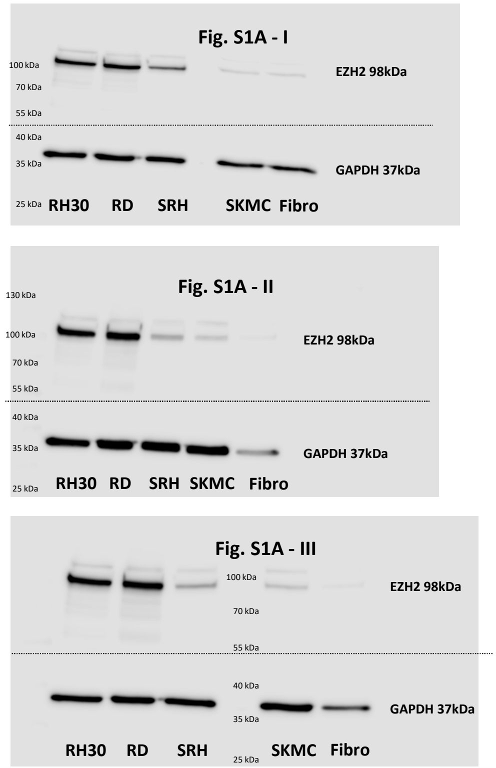

Figure S1B-F

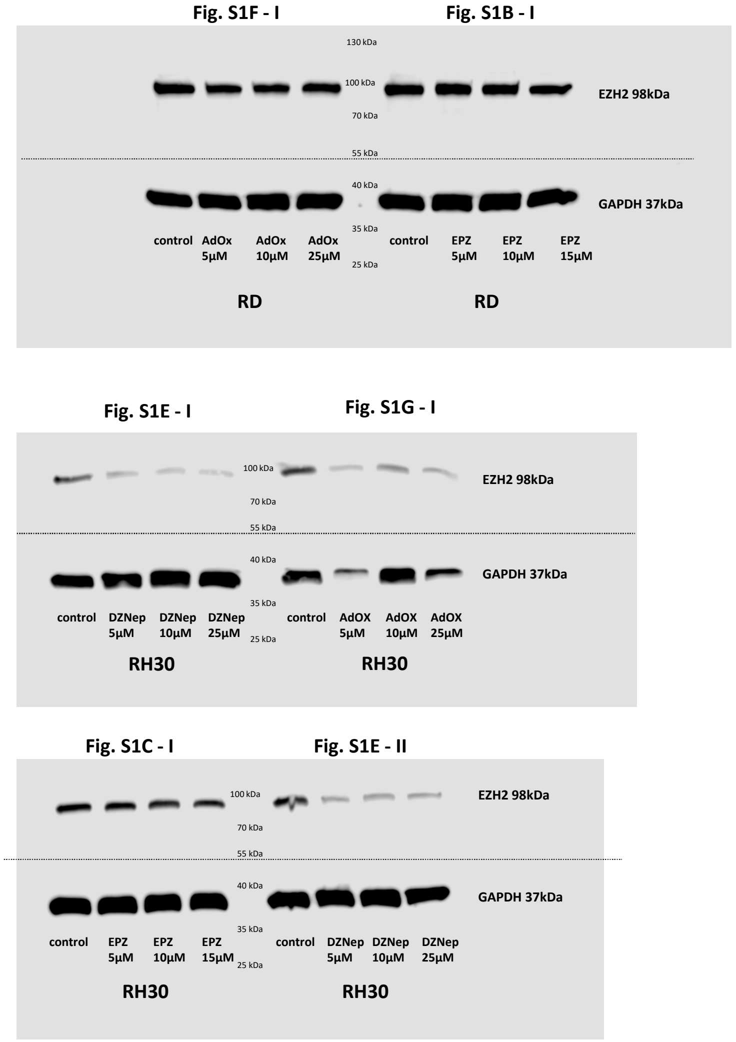

Fig. S1G - II Fig. S1C - II

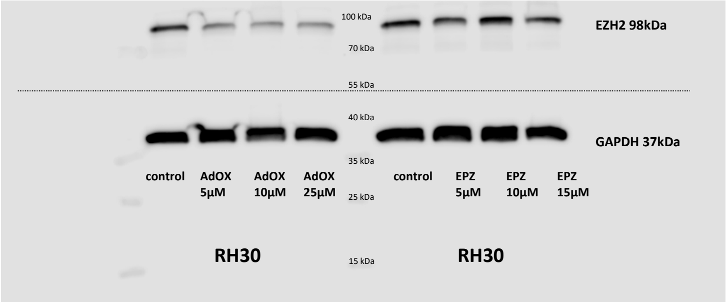

Fig. S1E - III Fig. S1G - III

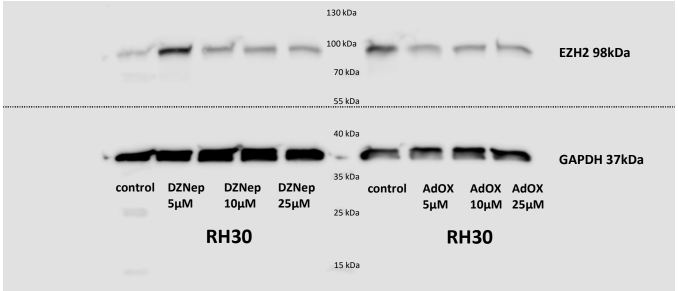

Fig. S1C - III Fig. S1D - I

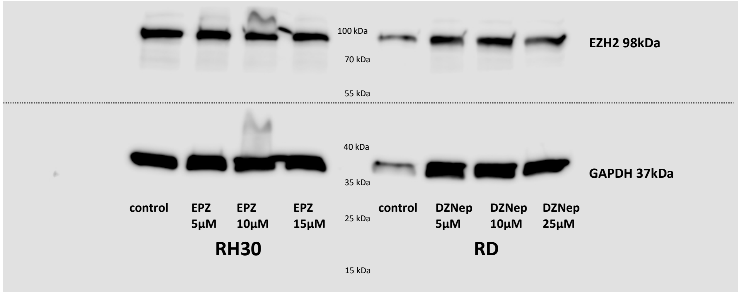

Fig. S1F - II Fig. S1B - II

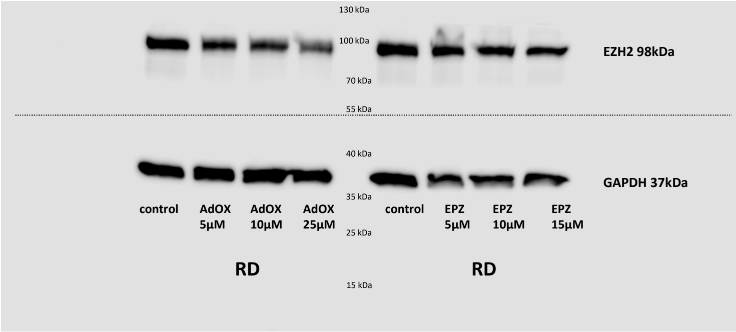

Fig. S1D - II Fig. S1F - III

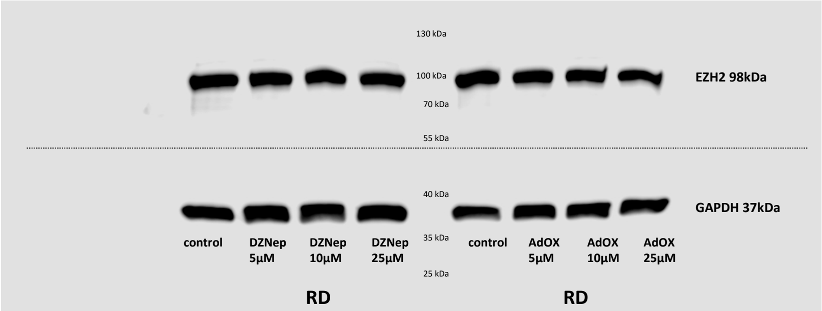

Fig. S1B - III Fig. S1D - III

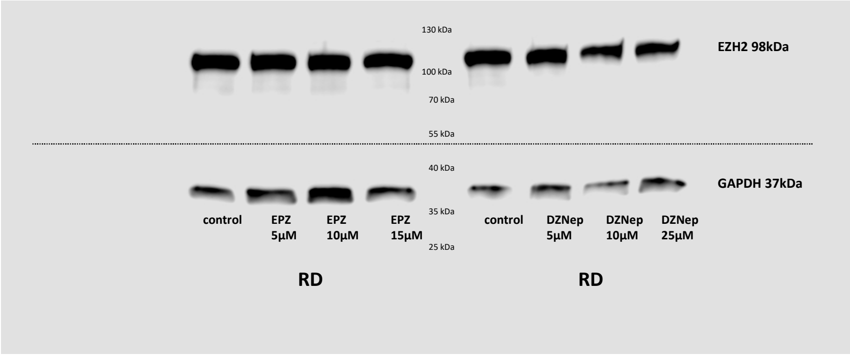

Fig. S1D – II – repeat **Fig. S1F – III – repeat**

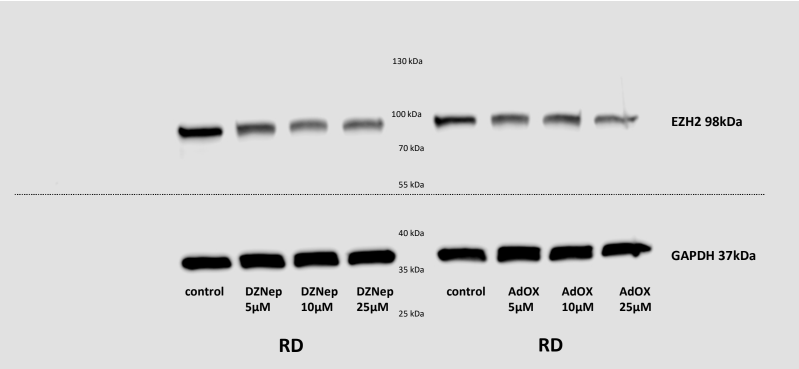

Fig. S1G - IV **Fig. S1D - IV**

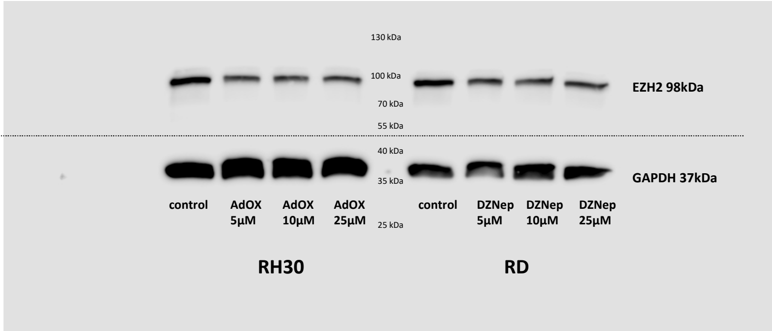

Fig. S1F – II – repeat **Fig. S1B – II – repeat**

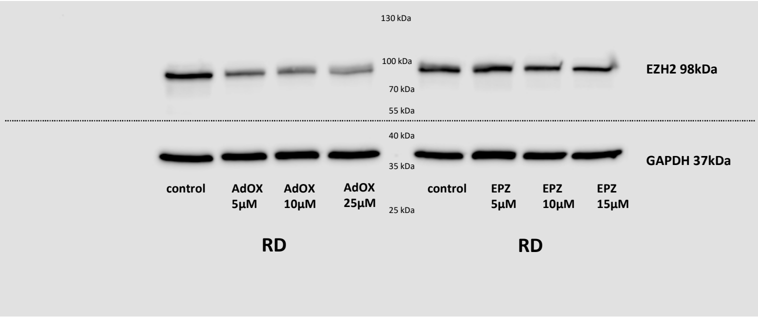

Fig. S1B – III – repeat **Fig. S1D – III – repeat**

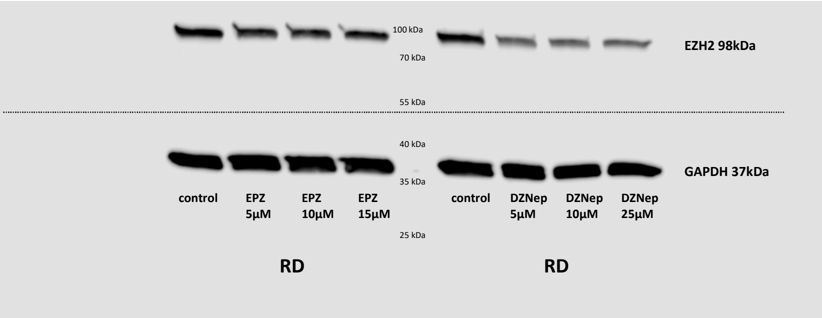

Supplement: Supplementary file 1 [file cancers-14-00041-s001.zip › cancers-1463800-supplementary.pdf]
